# The Depiction of Autonomy and Shared Decision-Making by Children and Adolescents in Medical Television

**DOI:** 10.7759/cureus.24662

**Published:** 2022-05-02

**Authors:** Marina E Golden, Ndifreke Ekpa, Bridget Rafferty, Robert P Olympia

**Affiliations:** 1 Pediatrics, Memorial Health University Medical Center, Children’s Hospital of Savannah, Savannah, USA; 2 Emergency Medicine, HCA Houston Healthcare Kingwood, Houston, USA; 3 Pediatrics, Janet Weis Children’s Hospital at Geisinger Medical Center, Danville, USA; 4 Emergency Medicine and Pediatrics, Penn State Hershey Medical Center, Hershey, USA

**Keywords:** autonomy, shared decision-making, assent, adolescents, children, medical television

## Abstract

Background

Many studies have shown the importance of patient autonomy and shared decision-making in medical treatment. However, television (TV) depiction of medicine continues to present a skewed depiction of healthcare and its effects. This has been observed in adult patients but little has been studied in the pediatric population.

Methodology

This study analyzed the depiction of pediatric patients (7-18 years old) autonomy and their participation in the shared decision-making process in the first season of medical TV dramas that premiered from 1994 to 2017, including *ER* (1994), *Grey’s Anatomy* (2005), *Red Band Society* (2014), and *The Good Doctor* (2017). These shows were scored to record each instance of a medical decision made.

Results

Of the 238 medical decisions recorded, pediatric patients made a medical decision 61 times (57.5%). A total of 110 instances were omitted due to the patient’s inability to give consent, usually due to altered mental status, and 22 instances were omitted due to age being less than seven years. Interestingly, there was an increasing proportion of pediatric patients involved in the decision-making process over time, moving from 17 of 39 medical decisions (43.6%) of patients in *ER* (1994) to 22 of 33 medical decisions (66.7%) in *The Good Doctor* (2017) (p = 0.050213).

Conclusions

The results revealed that TV medical dramas have been including children in their medical decision-making more over time. This has major implications for the way writers structure their shows and how medical providers interact with their patients.

## Introduction

The first medical television drama, *City Hospital*, premiered in the United States in 1951. Since then, such programs have become increasingly popular, with 50 new medical series premiering after 1990 [1]. At their peak, shows such as *ER* and *Grey’s Anatomy* reached, on average, 30.79 million and 19.44 million viewers per season, respectively [2].

The manner in which pediatric patients are depicted in mainstream media, like medical television dramas, can influence how patients and their families behave in real-life medical situations. The depiction of pediatric patients in popular media has evolved over time. The level of patient involvement and autonomy has fluctuated with the ever-increasing number of medical programs and their need to create drama. In the current cultural environment, it is important to examine the way patients are depicted in the media. Many viewers obtain healthcare knowledge from media; this change in characterization sparks a change in the way that patients and their families think and feel about healthcare [3,4].

Fictional medical media has an effect on “individuals’ knowledge about specific health topics, perceptions of healthcare and healthcare workers, and health behaviors” [5]. These perceptions are internalized and taken to outpatient clinics, emergency rooms, and surgical tables, resulting in serious adverse thoughts and outcomes for patients. Several studies have shown how depictions of disease and medical practice on TV have led viewers to incorrectly assume certain diseases are more prevalent in certain populations and that treatments are more effective than in reality [5]. These misconceptions can have detrimental effects both on patients’ utilization of healthcare and their understanding of their own health. Patients might delay seeking treatment, have a lack of trust in healthcare workers or the healthcare system, and feel an increase in overall fear and anxiety surrounding illness.

Patients’ perceptions become particularly important because of the recent shift toward patient-centered care by healthcare professionals. Patient-centered care has been endorsed as the premiere form of medical practice as it leads to better exchanging of information about healthcare treatments, more incorporation of the family’s preferences and values, and better health outcomes for patients [6]. All parties involved are generally happier with the service provided, regardless of the outcome, when healthcare providers, patients, and family members can discuss the best course of treatment, a process known as shared decision-making [2]. The process of shared decision-making generates unique challenges for the pediatric population due to issues of consent and assent for patients. Outdated guidelines and provider opinions have slowed the spread of pediatric involvement in their care. The American Academy of Pediatrics (AAP) suggests that “patients should participate in decision-making commensurate with their development; they should provide assent to care whenever reasonable” [7]. The United Nations further agreed that pediatric patients are capable of providing valuable insights into how they experience health and their healthcare [8,9]. For these reasons, we believe it is important to investigate the level of autonomy and shared decision-making depicted in popular media to begin the process of reviewing its effect on real-life pediatric patients. The goal of this endeavor is to encourage writers of medical programs to display a realistic idea of the level of involvement pediatric patients should have in their care and dissuade further spread of misconceptions.

In this study, we aimed to investigate the manner in which four selected series of medical TV dramas have depicted the pediatric patient’s degree of participation in shared decision making.

## Materials and methods

We conducted a retrospective, observational study starting in the fall of 2018. The first season of four medical dramas were analyzed. The series *ER* (1994), *Grey’s Anatomy* (2005), *Red Band Society* (2014), and *The Good Doctor* (2017) were chosen because they represent the most popular shows of their time periods in which children are frequently patients (further described in Table 1).

**Table 1:** Description of medical television series.

Title of series	Release year	Episodes	Description
*ER*	1994	25	Chronicles “the lives, loves, and losses of the doctors and nurses of Chicago’s County General Hospital” [10]
*Grey’s Anatomy*	2005	9	“A drama centered on the personal and professional lives of five surgical interns and their supervisors” [11]
*Red Band Society*	2014	13	“A group of teenagers live together as patients at a hospital’s pediatric ward and learn how to deal with their illnesses, the experiences that they have, and the people that they meet” [12]
*The Good Doctor*	2017	18	“Shaun Murphy, a young surgeon with autism and Savant syndrome, is recruited into the surgical unit of a prestigious hospital” [13]

Data acquisition

The data were recorded using a standardized data entry sheet created for this project. Data were independently compiled by three scorers. Each scorer was assigned to two of the four shows and asked to record instances of medical decisions, patient autonomy, diagnosis, procedures, as well as other relevant data points. Conflicts were resolved by consensus with clarification from a third scorer.

Medical decisions

A medical decision was recorded if a character (1) discussed or performed a decision on screen that (2) pertained to another character’s medical treatment, and (3) at least one party was of sound mind and capable of decision-making capacity.

Inclusion and exclusion criteria

Data were recorded for pediatric patients depicted in the show to be less than 18 years of age. A total of 132 instances of medical decision-making across the four series were omitted based on criteria that prevented informed consent and appropriate decision-making. These instances included patients who were seven years of age or younger due to the legal restrictions on consent and assent (22 instances) [6,14,15]. Similarly, patients were excluded from the study if they were incapable of giving informed consent in such situations as altered mental status, unconsciousness, or cognitive disabilities (110 instances).

Patient autonomy

Patients were determined to have autonomy in instances where they were informed of the medical options and given the opportunity to make a choice. Situations where patients were prevented from participating in the medical discussion or where their decision was disregarded were not classified as patient autonomy.

Shared decision-making

**Figure 1:**
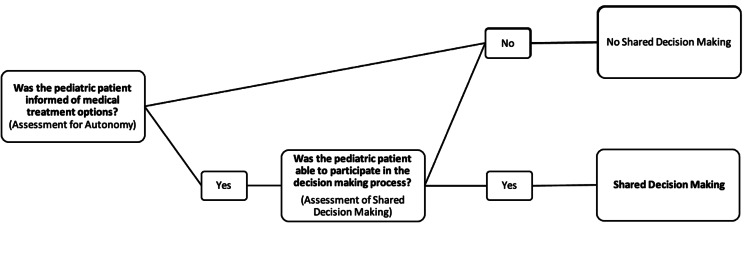
Schema for shared decision-making.

Instances of shared decision-making were aggregated based on instances in which patients were informed of a potential medical decision, were not prevented from making a decision by their guardian(s) or medical provider, and were able to assert their decision. This schema is outlined in Figure 1.

Data analysis

Data were recorded and stored in a Microsoft Excel document. The number of medical decisions was first stratified by the degree of patient autonomy and shared decision-making. Next, the frequencies of instances of autonomy and shared decision-making were compared to frequencies of no autonomy or shared decision-making by television series using the chi-squared test.

## Results

Of the 238 medical decisions recorded across the four series, pediatric patients were able to participate in the shared decision-making process 61 (57.5%) times, as depicted in Table 2. There was an increase in the percentage of medical decisions made by patients over time, with the greatest patient involvement occurring equally in *The Good Doctor* (2017) and *Grey’s Anatomy* (2005) at 66.7% and the least occurring in *ER* (1994) at 43.6%.

**Table 2:** Pediatric shared decision-making by series. *Percent shared decision-making = instances of pediatric shared decision-making/(Total instances of medical decision-making – instances omitted (consent) – instances omitted (age)).

Series	Total instances of medical decision-making	Instances omitted (consent)	Instances omitted (age)	Not informed of treatment options	Not able to assert decision	Instances of pediatric shared decision-making	Percent shared decision-making*
*ER*	133	77	17	9	13	17	43.6%
*Grey’s Anatomy*	20	14	0	0	2	4	66.7%
*Red Band Society*	35	7	0	7	3	18	64.3%
*The Good Doctor*	50	12	5	2	9	22	66.7%
Total	238	110	22	18	27	61	57.5%

**Figure 2:**
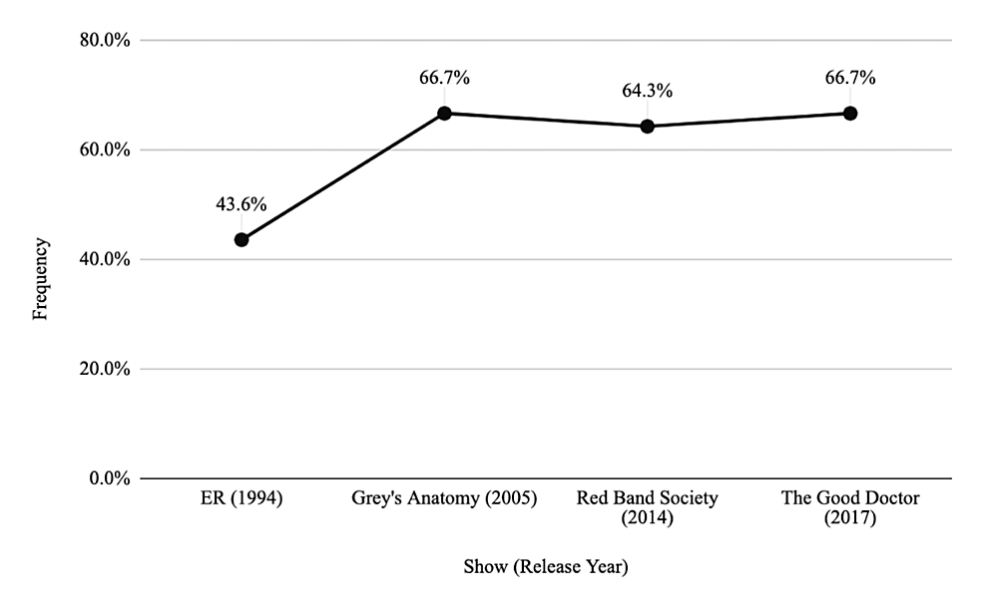
Frequency of shared decision-making by series over time.

There was an increasing proportion of pediatric patients involved in the decision-making process over time, moving from 17 of 39 medical decisions (43.6%) of patients in *ER* (1994) to 22 of 33 medical decisions (66.7%) instances in *The Good Doctor* (2017) (p = 0.050213). Figure 2 illustrates the large difference between decisions made and those involving shared decision-making between the series.

## Discussion

This study revealed that pediatric involvement in shared decision-making increased over time, but was not statistically significant. Despite an increase, this lack of statistical significance may lie in the small sample size of instances of medical decisions with pediatric patient involvement in the earlier shows, namely, *ER*. In the instances where pediatric patients were involved in earlier shows, patients were usually incapacitated and therefore unable to provide assent, consent, or involvement whatsoever in their medical care. In these cases, the data point was omitted from the study, resulting in a large proportion of instances omitted.

*ER*, for example, had far fewer instances of shared decision-making, which were exacerbated by the large number of medical decisions made on the show. This trend could be due to the greater support of shared decision-making by the pediatric and medical community at large over time. A possibility is that growing knowledge of the medical field, along with the empowerment of the general population to be involved in their medical care due to information propagation via the internet has led writers to depict patients similarly. Regardless, the results reveal that these shows are moving toward scripting patients who are more autonomous, more proactively involved in their healthcare, and more vocal about how they would like to be treated.

Some limitations of this study include the small sample size and ambiguity in the definition of a medical decision. The inclusion of a single season from each series yielded some with a few instances of medical decision making; *Grey’s Anatomy* only yielded six instances of medical decision making. *Red Band Society*, a show centered around a pediatric cancer ward, failed to produce a robust sample size. This sample may not be representative of all TV medical dramas. Thus, compiling data on additional series and/or additional seasons of the current series may be beneficial to future studies. Another limitation includes inter-rater reliability as there were discrepancies between scorer interpretation of scenes. This was resolved via consensus from a third scorer, but initial inter-rater reliability was not calculated.

Future directions for this study would also include an investigation into the impact that on-screen depictions of medical treatment have on the audience. For example, do these depictions affect patient relationships with medical professionals, cause families or patients to alter decisions about their own healthcare, or influence the audience in an entirely different way? Such studies to analyze the strength of patients on TV to influence real-life decision-making could be invaluable to the writers of these shows. This information could not only give a more realistic and, therefore, meaningful idea of what medicine can do and should be but also could be used by writers to disseminate information for the betterment of the healthcare field.

## Conclusions

The AAP and the United Nations have agreed that pediatric patients are capable of providing valuable insights into how they experience health and their healthcare. Increasingly, on-screen depictions show pediatric patients as knowledgeable, willing, and, most importantly, necessary participants in the medical decision-making process. This has implications for several real-life situations as patients and their families frequently develop their perceptions of healthcare through representations in the media. Our study revealed that pediatric patients depicted in TV medical dramas have been increasingly included in medical decision-making over time. This has major implications for writers’ structure of such shows and their depictions of medical providers interactions with patients. Physicians should be aware of such influences on patient beliefs and understanding of the healthcare they may receive. Furthermore, it is paramount to include pediatric patients in the medical decision-making process to the degree that it is appropriate based on age and decision-making capacity.
